# Variation in the degree of reciprocal herkogamy affects the degree of legitimate pollination in a distylous species

**DOI:** 10.1093/aobpla/ply022

**Published:** 2018-05-02

**Authors:** Xian-Feng Jiang, Xing-Fu Zhu, Qing-Jun Li

**Affiliations:** 1 Key Laboratory of Tropical Forest Ecology, Xishuangbanna Tropical Botanical Garden, Chinese Academy of Sciences, Mengla, China; 2 University of Chinese Academy of Sciences, Beijing, China; 3 Laboratory of Ecology and Evolutionary Biology, State Key Laboratory in Conservation and Utilization of Bioresources in Yunnan, Yunnan University, Kunming, Yunnan, China

**Keywords:** Disassortative pollination hypothesis, distyly, homostyly, pollen deposition, *Primula chungensis*, reciprocal herkogamy

## Abstract

Distyly is a widespread floral polymorphism characterized by the flowers within a population showing reciprocal placement of the anthers and stigma. Darwin hypothesizes that distyly evolves to promote precise pollen transfer between morphs. *Primula chungensis* exhibits two types of anther heights, and these two types of anthers show pollen of two different size classes. To understand whether the stigma could capture more pollen grains from the anthers of the pollen donor as the separation between the stigma of pollen receiver and the anther of pollen donor decreased, the present research assessed the source of the pollen load in a series of open-pollinated flowers with continuous variation of style lengths. Individuals with continuous variation of style length were tagged, and the selected flowers in the tagged plants were emasculated the day before dehiscence. The stigma of the emasculated flowers was fixed in fuchsin gel at the end of blooming. We assessed the pollen sources on each stigma by taking photos under a microscope and measured the diameter of each conspecific pollen grain with ImageJ. We found that a shorter distance from the stigmas to the anthers of a pollen donor gave the flower a higher capacity to receive pollen from those anthers. Our result provides a new evidence that distyly could promote the pollen transfer between morphs, which is consistent with Darwin’s hypothesis of disassortative pollination. An alternative hypothesis for the evolution of distyly (e.g. selfing avoidance) might also be true, but less likely, because self-incompatibility would greatly avoid self-fertilization for many distylous species.

## Teaser

Distyly, a well-known floral syndrome that was first identified by Charles Darwin, would be an ideal model to determine how a key floral syndrome is shaped by nature. *Primula chungensis*, a distylous species with simultaneous homo-, short- and long-styled morphs, exhibits some variations in the length of style. Using this species, we found that the stigma captured more compatible pollen as the separation between the stigma of the pollen receiver and the anther of the pollen donor decreased. Our results provide clear and novel evidence for the disassortative pollination hypothesis proposed by Charles Darwin, which will be helpful for our future understanding of the evolution of distyly.

## Introduction

Heterostyly is a classic floral polymorphism that is characterized by flowers showing reciprocal placements of male and female organs between two (distyly) or three morphs (tristyly) in a population (Ganders 1979; Barrett 1990; Lloyd and Webb 1992a; Barrett and Shore 2008; Barrett 2013). Distyly has two different floral morphs within a population, i.e. the long-styled morph and the short-styled morph. In long-styled morph, stigma is positioned higher than the anthers, whereas in short-styled morphs the reverse is there (Ganders 1979; Barrett 1990; Lloyd and Webb 1992a; Barrett and Shore 2008; Barrett 2013). The two floral morphs of most distylous species also differ from one another in terms of several floral traits. For example, the long-styled morph shows a wider corolla and shorter floral tube than the short-styled morph, and the pollen grains from the long-styled morph are significantly smaller than those of the short-styled morph. In addition, these two morphs also differ with respect to other traits such as pollen production, the width of the stigma and the length of the stigmatic papilla (Darwin 1877; Ganders 1976; Dulberger 1992; Ree 1997). Meanwhile, most distylous species is accompanied with a heteromorphic self-incompatibility system (SI system). Thus, pollination between different morphs is defined as ‘legitimate pollination’; in contrast, intra-flower and intra-morph pollination are known as ‘illegitimate pollination’.

Distyly has been known for a long time, and its evolutionary significance was first demonstrated by Darwin (1877) in his landmark book *The Different Forms of Flowers in Plants of The Same Species*, and he also wrote in his autobiography that ‘I do not think anything in my scientific life has given me so much satisfaction as making out the meaning of the structure of heterostylous flowers’ (Darwin 1887). According to his hypothesis, the reciprocal herkogamy of distyly improves the efficiency of pollen movement between the flowers of long- and short-styled morphs, thus decreasing the probability of pollen transfer between flowers of the same flower or morph. Based on the disassortative pollination hypothesis of Darwin, Lloyd and Webb (1992a, b) assumed that distyly evolves from a monomorphic ancestor and develops reciprocal herkogamy for the purpose of promoting inter-morph pollen transfer. Whereas Charlesworth hypothesized that the pressure of avoiding self-fertilization leads to the selection of distyly (Charlesworth and Charlesworth 1979a, b). Various evolutionary biologists questioned that it is redundant for the distylous species to develop two different syndromes for the same purpose, and are in a consistent with the disassortative pollination hypothesis (Pérez-Barrales *et al.* 2006). Some studies examined the disassortative pollination hypothesis using manipulating experiment (Keller *et al.* 2014) or molecular methods (Zhou *et al.* 2015). Most studies quantified disassortative pollen movement by analysing pollen deposition on stigmas under natural conditions (Ganders 1976; Ornduff 1979; Nishihiro *et al.* 2000; Valois-Cuesta *et al.* 2011; Liu *et al.* 2015).

**Figure 1.**
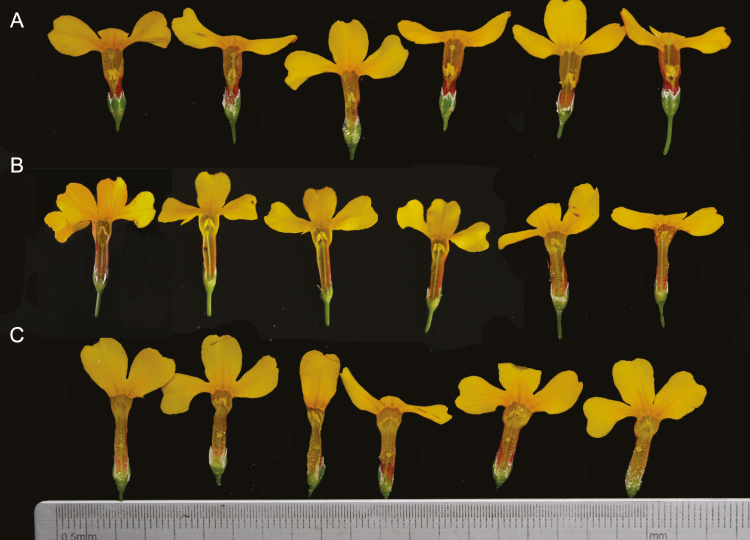
Flowers from the three *Primula chungensis* morphs showing slight variation in the length of the style were collected in the field. The sketch shows (A) the flowers from the long-styled morphs, (B) the flowers from the homostylous morphs and (C) the flowers from the short-styled morphs. The variants from homostylous morph to (B) short-styled morph (C) were selected for our manipulative experiment.

**Figure 2.**
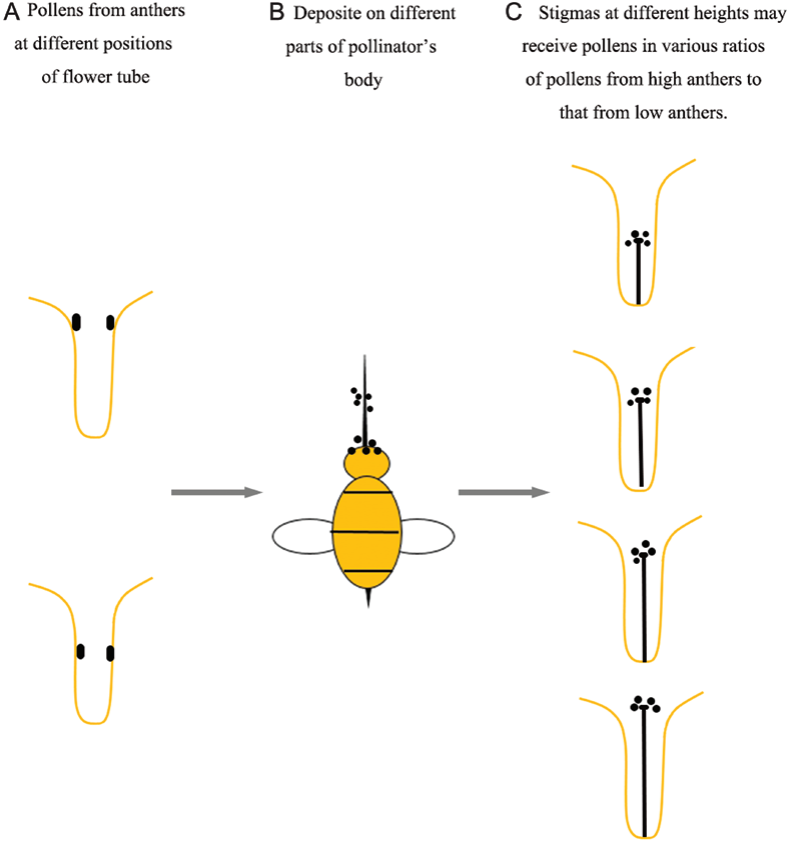
Sketches of this manipulative experiment are displayed. (A) *Primula chungensis* flowers were grouped into two types based on the heights of the anthers, and the pollen grains found on the anthers with different heights differ significantly in size. (B) The pollen grains of the anthers with different heights adhered to different parts of the body of the pollinator. (C) The flowers utilized in this experiment varied in style length; thus, we used the emasculated flowers as the pollen receptors. We expected to see a correlation between the proportion of compatible pollen and the length of the style.

Distyly is one of the most effective floral syndromes for avoiding self-fertilization, but it can also frequently break down into self-compatible homostylous morph (de Vos *et al.* 2014). *Primula* is a genus in which the majority of species exhibit a typical distylous syndrome, and a self-compatible homostylous species is also commonly reported to occur in this genus (Richards 2003). Same primroses possess both distylous and homostylous morphs, in which the homostylous morph is found to be similar to the short-styled morphs in terms of some floral characters such as pollen production (Piper *et al.* 1986) and pollen size (Piper and Charlesworth 1986). *Primula chungensis* has three morphs, and slight variations in the lengths of the styles occur in all three morphs of *P. chungensis* in natural habitats (Fig. 1). Thus, we used some individuals of short-styled and homostylous morphs that continuous vary in the length of the style to conduct the experiment. The selected flowers were emasculated, and the style length variation was used to assess whether flowers could capture more pollen grains as the stigma of pollen receptor more closely approaches the position of the anthers of the pollen donors (Fig. 2). According to the evolutionary model developed by Lloyd and Webb, inter-morph pollen transfer should reach its maximum when the anthers and stigmas of reciprocal morphs are perfectly matched (Lloyd and Webb 1992a, b). If the disassortative pollination hypothesis is true, we expect to see that the component of inter-morph pollen transfer would increase as the stigmas and anthers of opposite morphs become of increasingly similar size.

## Methods

### Study species and site

*Primula chungensis* is a perennial herb belonging to section *Prolifera* of *Primula* (Richards 2003). Based on our measurements in the field, *P. chungensis* has an average of 22.41 flowers per inflorescence and are separated into different whorls, and each inflorescence generally shows 3–5 whorls. *Primula chungensis* flowers from late April to June, with each single flower generally blooming for 4–6 days. We observed *P. chungensis* for 8 h during the peak flowering season in 2015, and found that *P. chungensis* was mostly visited by *Bombus richardsi*, and was occasionally visited by *B. convexus*, *B. atrocictus* and a species in the genus *Rhingia* of Syrphidae. *Primula chungensis* is distributed throughout south-eastern Tibet, western Sichuan Province and north-western Yunnan Province, China (Richards 2003). An experiment was performed in a population near the Southeast Tibet Ecological Station, Chinese Academy of Science (henceforth ES) in which more than 10000 individuals of the three morphs were distributed (N29°33.675′; E94°44.675′, 3328 m a.s.l.). In 2014, we investigated the morph ratios of this population by randomly selecting 1821 individuals, of which were 416 homo-styled morph, 636 short-styled morph and 769 long-styled morph. The flowers with the three *P. chungensis* morphs varied slightly in the lengths of their styles (Fig. 1 and **Supporting Information—Table S1**).

### Characterizing the variations in pollen size and anther height as well as the consistency of the style length

To understand whether or not the sizes of pollen grains from *P. chungensis* differed significantly among the three morphs, pollen grains from the three morphs were scanned with a scanning electron microscope (SEM). The pollen samples were collected from a newly opened flower and preserved in 70 % ethanol solution, and then were subjected to a gradient dehydration with 70 %, 95 % and absolute ethyl alcohol. Fully dehydrated pollen samples were sprayed with gold before being imaged with the SEM at ×5000 magnification. In addition, to understand the frequency distribution of the *P. chungensis* pollen sizes in the tri-morph population, we measured the diameters of the pollen grains of *P. chungensis* under a light microscope. For each morph, one non-dehiscent flower bud was randomly collected from each of 10 individuals. Pollen grains of the same morph were mixed sufficiently in a small tube (1 mL) with 70 % ethanol solution. Then, 5 µL of pollen mixture from each morph was pipetted onto a glass slide and then observed under the microscope at ×400 magnification. The pollen grains from each morph were imaged, and the diameter of each pollen grain in the images was measured using ImageJ 4.0. In total, 150 pollen grains from each morph were measured.

**Figure 3.**
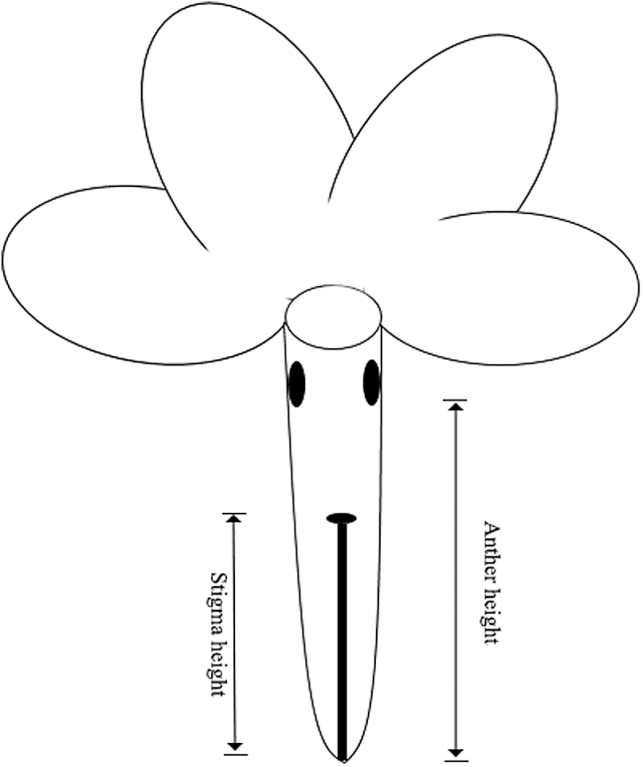
Sketch shows the two floral traits measured in the experiment. Anther height was measured from the basal flower tube to the lowest point of the anthers, and stigma height was that from the basal flower tube to the stigma.

To understand how the anther heights in *P. chungensis* were distributed in the ES population, one fully blooming flower from a single individual was randomly collected from a total of 79 flowers with short-styled morphs, 79 flowers with long-styled morphs and 65 flowers with homostylous morphs. Anther height was measured from the basal corolla to the lowest point of the anther in the flower (Fig. 3). We drew a frequency distribution plot of the heights of the anthers and the sizes of *P. chungensis* pollen to see how the distributions of the two morphological traits varied in a natural habitat. The normality of the collected data was tested using the UNIVARIATE procedure and the data fitting normal distribution were compared using ANOVA; the other data were compared with a non-parametric test (GLIMMIX procedure). We compared the pollen sizes and anther heights among the three morphs by one-way ANOVA, with GLIMMIX procedure. When the difference was significant, a *post hoc* Tukey test was conducted to assess the differences among the treatments. *t*-Test was applied to evaluate the statistical significance of the difference between high anthers and low anthers, as well as that between small and large pollen. All the data in this paper are reported as the means ± SE, and statistical analyses we used were performed in SAS version 9.2.

To understand whether the length of the style was consistent throughout the whole anthesis period of a flower, we randomly tagged one flower bud from each single individual, for a total of 10 flower buds to be measured from each morph. The lengths of the styles and the heights of the anthers were measured daily from the basal corolla to the stigma (Fig. 3) from the first blooming day until the corollas naturally withdrew, and each tagged flower was measured repeatedly for 4–5 times. Using repeated-measures ANOVAs with the GLM procedure, we examined the significance of differences along the time series for single flowers at five time points starting in the data from the first day of anthesis. Most measured flowers faded on the fifth day. However, four flowers of the homostylous morph, three flowers of the short-styled morph and one flower of the long-styled morph faded on the fourth day. Therefore, in the repeated-measures ANOVA, we considered the values for the fifth blooming day of these flowers as missing. There were two factors in this analysis, i.e. the morph and the date, and we assigned the ‘morph’ as the random effect and simply checked the statistical significance of the ‘date’. To examine whether the style length and anther height varied markedly among the flowers on different positions of an inflorescence, stigma heights and style lengths of the flowers from eight inflorescences were measured and compared among the basal, middle and distal positions. This data set was tested via the GLIMMIX procedure.

### Test of the disassortative pollination hypothesis

Although the flowers of the long-styled morph displayed slight variation in the length of style, we did not found continuous variation from the long-styled morph to the homostylous morph (Fig. 2). The floral shape was also significantly different between the long- and homostylous morphs (corolla width and floral tube length) (Jiang *et al.* 2017). Meanwhile, manipulation of the long-styled morph (emasculated treatment) would inevitably destroy the flower tube, which may affect the pollinator visitation. Thus, we did not perform the manipulated experiments using the flowers of long-styled morph.

**Figure 4.**
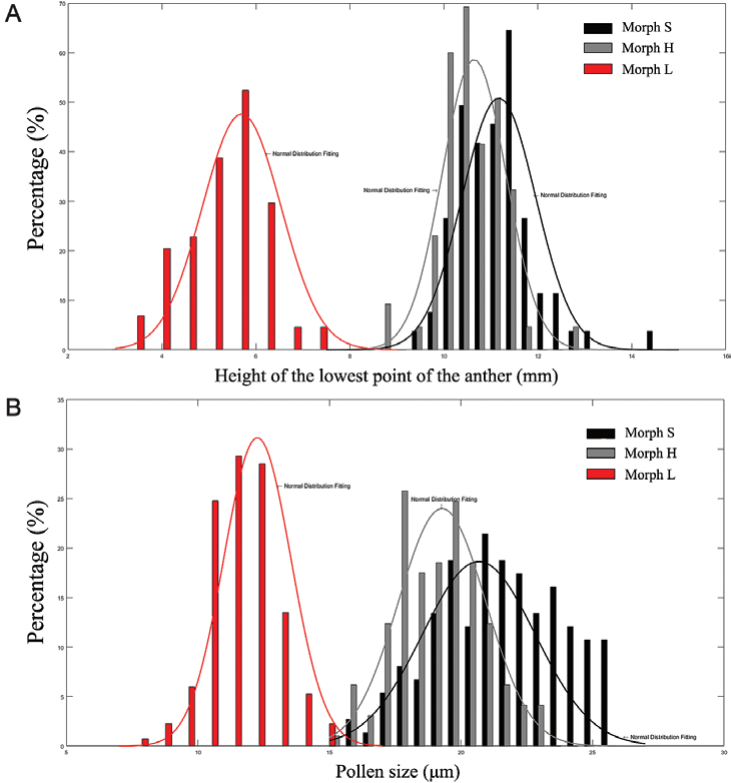
Frequency distribution of (A) the height of the anther and (B) the size of the pollen grains from *Primula chungensis* in natural habitat. The curve in each plot shows the expected normal distribution of each data set.

At the beginning of the flowering season in 2015, we marked 22 individuals of the homostylous morph and 18 individuals of the short-styled morph **[see****Supporting Information—Table S1****]** that differed sequentially in the lengths of their styles. To eliminate potential measurement errors, the style lengths of each inflorescence were determined by randomly repeating the measurements on three fully blooming flowers according to the criteria in Fig. 3. To examine whether the pollen source was markedly affected by the position of the stigma, two flowers from each tagged inflorescence were randomly selected for the use in manipulative experiment. Before blossoming, the selected floral buds of each tagged inflorescence were emasculated by carefully removing the anthers from the mouth of each flower tube with a forceps; a total of 80 flowers were emasculated. Although we were unable to determine the effects of intra-flower pollen deposition on illegitimate pollination with the emasculated flowers, this approach could clearly indicate the inter-flower pollen transfer. Each single *P. chungensis* flower generally blooms for 4–6 days, and on the sixth day, we collected the stigmas of the emasculated flowers. The pollen grains that were deposited on the stigmas were examined using fuchsin gel according to Kearns’ method (Kearns and Inouye 1993), which has been applied to a large number of species, including *Delphinium barbeyi* (Briggs *et al.* 2015) and *Euphorbia esula* (Montgomery and Rathcke 2012). First, gel that had been dyed pinkish-red was picked up with a forceps, laid out on a glass slide and then melted with a lighter. Second, the stigmas of the tagged individuals were carefully picked up and laid on the melted gel after repeatedly touching the melted gel several times in order to help the pollen to spread on the melted gel as far as possible, and then the cover glass was added. Fourteen flowers were destroyed before we collected the stigmas, and an additional eight samples were tagged to compensate for their loss. In the end, a total of 33 stigmas from the short-styled morph and 43 stigmas from the homostylous morph were collected and brought back to our laboratory. We observed the pollen grains on each stigma by taking images under microscope at ×400 magnification, and determined whether a pollen grain belonged to *P. chungensis* based on its size and shape. The diameter of each *P. chungensis* pollen grain was measured in ImageJ 4.0. Because *P. chungensis* underwent severe pollen limitation in the ES population during 2015 flowering season, half of the collected samples had no pollen deposited on them. However, this limitation facilitated our ability to count the number of pollen grains. For the nine samples loaded with a large number of pollen grains (more than 200 pollen grains), we carefully separated each grain in the image with a circle and then measured it in ImageJ. To eliminate the influence of occasional events that led to minimal pollen deposition, stigmas with a deposition of over 10 pollen grains were used in the correlation analysis between the length of the style and the pollen source. To that end, 37 samples were used in the analyses to determine the correlation between the length of the stigma and the pollen load. The sizes of the pollen grains of short-styled and homostylous morphs differed markedly from that of the long-styled morph, and the least amount of overlap between the small pollen (pollen of the long-styled morph) and large pollen (pollen of the short-styled and homostylous morphs) was at 15.34 μm (Fig. 4B). Therefore, we designated the pollen grains that were smaller than 15.34 μm as ‘small’ pollen and grains larger than 15.34 μm as ‘large’ pollen. To prevent the small overlap between the ‘large’ and ‘small’ pollen grains from affecting our division results, the numbers of pollen grains that moved from ‘high’ or ‘low’ anthers were calculated from the numbers of observed ‘large’ and ‘small’ pollen grains using the following formulas as proposed by Ganders (1976):

$$Ti=\frac{\;Si-a\;(Li\;+\;Si)}{b-a}$$

and

Pi=Li+Si−Ti

where *Ti* = the number of pollen grains from high anthers in sample *i* (anthers of homostylous and short-styled morphs), *Pi* = the number of pollen grains from low anthers in sample *i* (anthers of the long-styled morph), *Li* = the number of pollen grains larger than 15.336 μm in sample *i*, *Si* = the number of pollen grains smaller than 15.34 μm in sample *i* (*Li* and *Si* > 0), *a* = the frequency of pollen grains from long-styled morphs that were smaller than 15.34 μm in the size distribution of measured small pollen (99.67 %) and *b* = the frequency of pollen grains from short-styled and homostylous morphs that were smaller than 15.34 μm in the size distribution of measured small pollen (0.67 %). The numbers of pollen grains from high and low anthers were calculated on a per-stigma basis, and the proportions of pollen from the high and low anthers in each sample were determined by dividing the number of conspecific pollen grains, i.e. *Ti* + *Pi*. Since the data did not follow a normal distribution, and the sample size was relatively small (*n* = 37), the correlation analyses were performed by using Spearman rank correlations (PROC CORR).

## Results

### Characterizing the variation in the pollen size, anther height and the consistency of the style length

**Figure 5.**
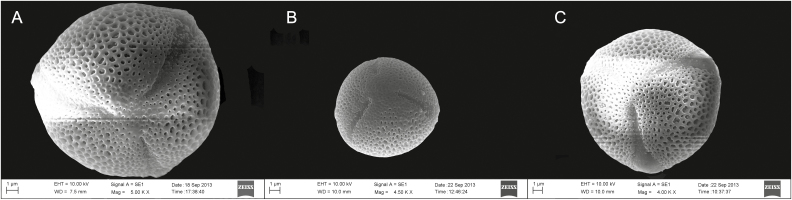
The pollen grains from the three *Primula chungensis* morphs under SEM at ×5000, (A) pollen from homostylous morphs, (B) pollen from long-styled morphs and (C) pollen from short-styled morphs.

The anthers of long-styled morphs were significantly lower than the anthers of short-styled and homostylous morphs, and anther position did not show obvious differences between homostylous and short-styled morphs (anthers of homostylous morphs: 10.64 ± 0.08 mm, anthers of short-styled morphs: 11.14 ± 0.09 mm and anthers of long-styled morphs: 5.60 ± 0.09 mm***, *F*_2, 222_ = 414.12, *P* < 0.0001). The pollen from long-styled morphs was strikingly smaller than that of the other two morphs (Figs 4B and 5), and the sizes of the pollen grains from homostylous and short-styled morphs were not significantly different (pollen of homo-styled morph: 19.28 ± 0.13 μm, pollen of long-styled morph: 12.26 ± 0.10 μm*** and pollen of short-styled morph: 20.68 ± 0.17 μm, *F*_2, 449_ = 8.89, *P* = 0.0029). Pollen size of *P. chungensis* in population ES could be easily grouped into two categories (small pollen: 12.26 ± 0.10 μm versus large pollen: 19.98 ± 0.12 μm, *T* = 59.93, *P* < 0.0001, *N* = 450), and the same was there for the anther height (high anthers: 10.91 ± 0.07 mm versus low anthers: 5.60 ± 0.09 mm, *T* = 51.27, *P* < 0.0001, *N* = 223) (Fig. 4).

No significant difference was observed in the length of the style throughout the whole anthesis of a single flower (first day: 10.12 ± 0.42 mm, second day: 10.12 ± 0.43 mm, third day: 10.08 ± 0.42 mm, fourth day: 10.11 ± 0.42 mm and fifth day: 10.2 ± 0.50 mm; *F*_4, 139_ = 0.87, *P* = 0.4839,) or among the flowers in different positions within the inflorescence (basal flowers: 9.80 ± 0.35 mm, middle flowers: 9.56 ± 0.23 mm and distal flowers: 9.73 ± 0.36 mm; *F*_2, 162_ = 0.01, *P* = 0.9227).

### Test of the disassortative pollination hypothesis

**Figure 6.**
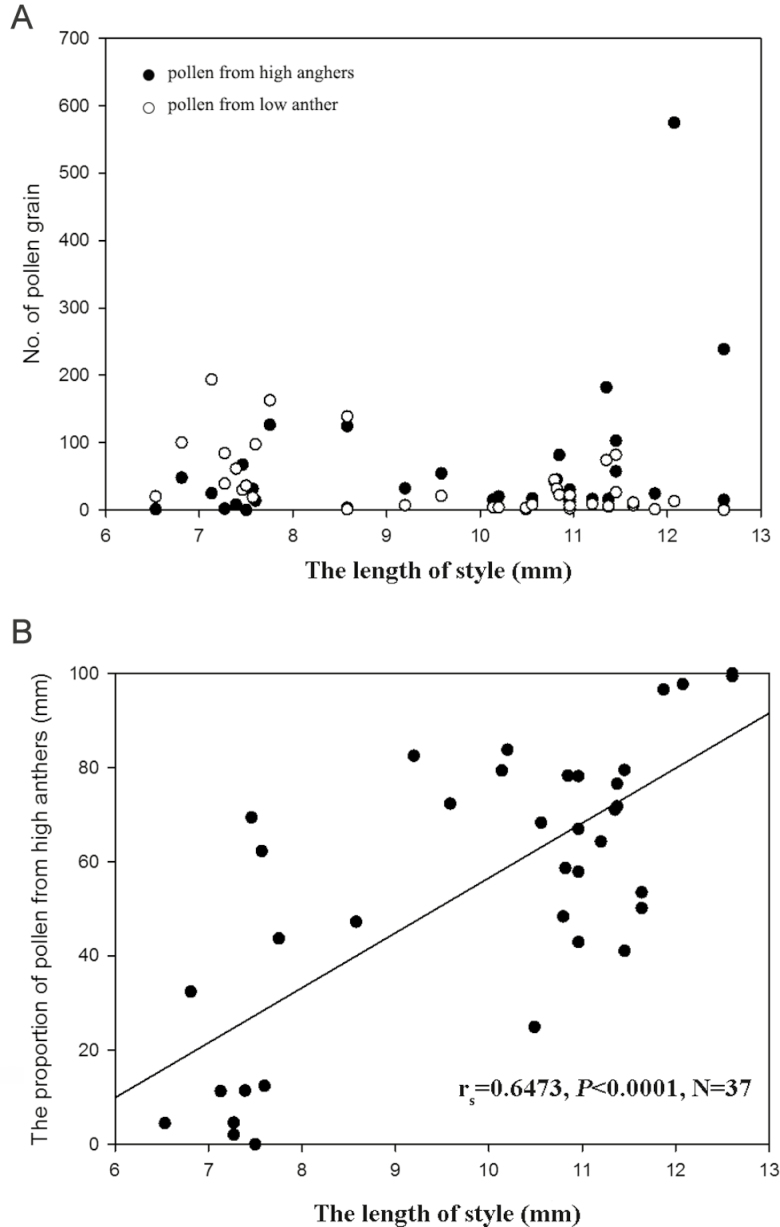
(A) The pollen grains of *Primula chungensis* mostly deposit on the stigmas that approach the anther height of the pollen donors; (B) the percentage of the pollen from the high anthers was significantly positively correlated with the length of the style of *P. chungensis*.

Manipulated flowers possessed style lengths that varied continuous from 6.94 to 12.94 mm. In total, 2105 large pollen grains, 1448 small pollen grains and 2173 heterospecific pollen grains were found in our samples. After correction using the Ganders’ model (Ganders 1976), 2114.5 large pollen grains from high anthers and 1438.5 small pollen grains from low anthers were counted. A positive but non-significant relationship was observed between the style length and the proportion of heterospecific pollen deposition (*r*_s_ = 0.1931, *P* = 0.2522, *N* = 37). Most conspecific pollen grains were deposited on the stigmas that matched the anther position of the pollen donors (Fig. 6A). The length of the stigma was significantly positively correlated with the proportion of pollen from the high anthers (*r*_s_ = 0.6473, *P* < 0.0001, *N* = 37) (Fig. 6B).

## Discussion

Distyly is a prevailing stylar polymorphism that is found in at least 28 families and has been reported and investigated for more than a century (Barrett and Shore 2008). Revealing the functional significance of distyly may help us to understand why an essential floral syndrome is selected in the field. The present study focused on the functional significance of distyly from the perspective of disassortative pollination hypothesis. We attempted to verify this hypothesis by selecting *P. chungensis* flowers that showed continuous variation in style lengths. We found that most conspecific pollen grains were deposited on stigmas that were similar in height to the anther height of pollen donors (Fig. 6A). Our result also revealed that the stigma had more compatible pollen grains deposited as its style height approached the anther height of pollen donor (Fig. 6B). Therefore, our experiment provided an evidence for the disassortative pollination hypothesis that distyly promotes more precise pollen transfer between morphs when there is close reciprocal matching of anther and sigma height.

A series of floral traits such as the corolla width, flower tube length and pollen size are reported to differ between the morphs of a distylous species. In the distylous primrose that is also with homostylous morph, the homostylous morph is similar to the short-styled morph in various flower traits (Piper *et al.* 1986; Piper and Charlesworth 1986). In *P. chungensis*, the homo-styled morph is similar to the short-styled morph in pollen production and anther height, and the pollen grains from the two types of anthers also differed significantly from each other in size (Fig. 4). Those characters could help us easily to distinguish whether the deposited pollen comes from the high anthers or low anthers.

According to the disassortative hypothesis, pollen grains from the anthers in different positions in a flower are likely to be attached to different parts of a pollinator’s body and thus transferred to the stigmas of opposite morphs (Olesen 1979; Wolfe and Barrett 1987; Brys *et al.* 2008). Many approaches, such as using man-made flowers as the pollen receptor (Stone and Thomson 1994), manually changing the herkogamy of the flower (Liu *et al.* 2015), or controlling the pollinator when it visits the flowers of cultivated plants (Keller *et al.* 2014) have generally supported the hypothesis, but these approaches might affect the pollinator’s behaviour, such as the duration per visit and the foraging preference. Meanwhile, notably more pollen grains were deposited on the stigma when a controlled pollinator visited the flower. For instance, Piper *et al.* (1986) found averages of 258.8 and 256.1 legitimate pollen grains on intact flowers from *P. vulgaris* long- and short-styled morphs under natural conditions, but Keller *et al*. (2014) found that almost 1000 and 400 legitimate pollen grains were deposited per stigma in long- and short-styled morphs of *P. vulgaris* when controlling the bumblebee during its visit to the flowers.

We used the continuous variation in style length of *P. chungensis* flowers in the field to examine the disassortative pollination hypothesis. Our results indicated that most conspecific pollen grains were deposited on the stigmas as they approached the height of anthers of the pollen donors (Fig. 6A). Meanwhile, we found that the length of the style showed a positive correlation with the percentage of pollen from the high anthers (Fig. 6B), which indicated that shortening the style would increase the proportion of pollen from low anthers and that elongating the style would increase the proportion of pollen from high anthers, suggesting that a stigma can more easily capture pollen from the anthers of the opposite morph as both of them approach the same height.

The disassortative pollination hypothesis refers to inter-flower pollen transfer; thus, an emasculated experiment is essential for testing this hypothesis. For example, Lau and Bosque (2003) found support for the disassortative pollination hypothesis using emasculated flowers. However, some studies aiming to test the disassortative pollination hypothesis found only weak support for the hypothesis because they studied stigmatic pollen loads only in intact flowers (Hernández and Ornelas 2007; Brys *et al.* 2008). Meanwhile, using only intact flowers in the studies would also cause low legitimate pollen deposition. For example, the legitimate pollen constituted 12.7 and 39.2 % of the pollen loads in intact flowers (Ornduff 1979), while 39 and 67 % of those in emasculated flowers (Piper and Charlesworth 1986) in the long- and short-styled morphs of *Primula vulgaris*. Although our study lacked an experimental series that included intact flowers, and we were thus unable to assess the contribution of pollen load sized from intra-flower selfing, our approach using emasculated flowers provided clear results that shortening the separation between the stigma of pollen receiver and the anther of pollen donor could significantly increase the proportion of inter-flower pollen transfer. We clearly prove that reciprocal placements of anthers and stigma could significantly promote pollen transfer between morphs, which is the core view of the disassortative pollination hypothesis proposed by Darwin.

Distyly is one of the most effective floral polymorphisms for promoting cross-fertilization. However, distyly is evolutionarily unstable in the field, and is frequently reported to breakdown to self-compatible homostyly (de Vos *et al.* 2014) or to evolve to dioecism (Ornduff 1966). The homostylous flowers of *P. chungensis* co-occur with distylous flowers in the same population, indicating that *P. chungensis* probably undergoes an evolutionary transition between self-fertilized homostyly and cross-fertilized distyly. The research based on phylogenetic and phylogeography methods revealed that *P. chungensis* is evolving from distyly to homostyly (Zhou *et al.* 2017). The present study indicated that the reciprocal herkogamy of distylous species could promote inter-flower pollen transfer as the positions of anthers and stigma become more similar in height, while the homostylous morph also has functional significance in the field because it can ensure its reproduction via autonomous self-fertilization (Jiang *et al.* 2017).

## Conclusion

The conspecific pollen load was mostly deposited on stigmas that matched the positions of the anthers of the pollen donors and that the compatible pollen source was highly correlated with the length of the flower style, indicating that shortening the distance between the stigma height and the height of the anthers of pollen donor would increase the received percentage of pollen from those anthers.

## Sources of Funding

This research was supported by the Joint Funds of National Science Foundation of China and the Yunnan Provincial Government (no. U1202261); the National Natural Science Foundation of China (no. 31500194); the CAS 135 programme (XTBG-F01, T01).

## Contribution by the Authors

X.-F.J.: field experiment, data analysis and manuscript preparation; X.-F.Z.: field experiment; Q.-J.L.: basic scientific idea.

## Conflict of Interest

None declared.

## Supporting Information

The following additional information is available in the online version of this article—

**Table S1**. Original data for the pollen deposition on 76 *Primula chungensis* stigmas.

Supporting Information
